# Primary and Secondary Prevention of Ischemic Stroke in Elderly Patients with Cardiovascular Disease: The Role of Frailty and Care Pathways

**DOI:** 10.3390/neurolint18020036

**Published:** 2026-02-14

**Authors:** Fabiana Lucà, Roberto Ceravolo, Michele Massimo Gulizia, Sandro Gelsomino, Carmelo Massimiliano Rao, Nadia Ingianni, Giuseppina Vitale, Giovanna Geraci, Attilio Iacovoni, Pietro Scicchitano, Adriano Murrone, Claudio Bilato, Luigina Guasti, Furio Colivicchi, Fabrizio Oliva, Federico Nardi, Massimo Grimaldi, Iris Parrini

**Affiliations:** 1 Cardiology Department, Grande Ospedale Metropolitano of Reggio Calabria, 89124 Reggio Calabria, Italy; fabiana.luca92@gmail.com; 2 Cardiology Unit, Giovanni Paolo II Hospital, 88046 Lamezia Terme, Italy; 3 Cardiology Department, Garibaldi Nesima Hospital, 95122 Catania, Italy; 4 Cardiothoracic Department, Maastricht University Hospital, 6229 Maastricht, The Netherlands; 5 Cardiology Unit, Santa Maria Degli Ungheresi Hospital, Polistena, 89024 Reggio Calabria, Italy; 6 Cardiologic District, Azienda Sanitaria Provinciale Trapani, 91016 Trapani, Italy; 7 Neurology Department, ARNAS Garibaldi Hospital, 95124 Catania, Italy; 8 Cardiology Department, Sant’Antonio Abate Hospital, Erice, 91016 Trapani, Italy; 9 Department of Transplant Surgery and Surgical Treatment of Heart Failure, Cardiovascular Department, ASST Papa Giovanni XXIII, 24127 Bergamo, Italy; 10 Cardiology Section, Hospital “F. Perinei”, Altamura, 70022 Bari, Italy; 11 Cardiology, Citta di Castello Hospital, 06012 Citta di Castello, Italy; 12 Cardiology Department, Ovest Vicentino Hospitals, Azienda ULSS 8 Berica, 36100 Vicenza, Italy; 13 Department of Medicine and Surgery, University of Insubria, 21100 Varese, Italy; 14 Clinical and Rehabilitation Cardiology Department, San Filippo Neri Hospital, ASL Roma 1, 00135 Roma, Italy; 15 Cardiology Unit, ASST Grande Ospedale Metropolitano Niguarda, 20162 Milano, Italy; 16 Cardiology Department, Santo Spirito Hospital, Casale Monferrato, 15033 Alessandria, Italy; 17 Cardiology Department, General Regional Hospital “F. Miulli’’, Acquaviva delle Fonti, 70021 Bari, Italy; 18 Cardiology Unit, Koelliker Hospital, 10134 Torino, Italy; irisparrini@libero.it

**Keywords:** stroke, cardiovascular diseases (CVD), elderly patients, secondary prevention, multidisciplinary approach, frailty, secondary prevention, frailty, atrial fibrillation, oral anticoagulation, LDL cholesterol, integrated care pathways

## Abstract

Stroke is a major global health concern, particularly among the elderly, who frequently present with multiple comorbidities, most notably cardiovascular diseases. Importantly, atrial fibrillation confers a nearly fivefold increase in stroke risk and accounts for up to one-quarter of ischemic strokes in older adults. Stroke is a neurological disease characterised by a strong cardiovascular interplay, and its multifactorial nature requires an integrated preventive approach. This review focuses on primary and secondary prevention in this population, with a frailty-informed perspective. We synthesise evidence on blood pressure control, lipid-lowering (including LDL-C targets), glycemic management, and antithrombotic strategies—particularly oral anticoagulation for atrial fibrillation—as well as the role of frailty indices in guiding individualised risk–benefit decisions. We also discuss practical care pathways, including structured post-discharge programs, continuity of care, and the need for multidisciplinary collaboration involving cardiologists, neurologists, and primary care. We highlight how frailty indices refine risk–benefit assessments without justifying therapeutic nihilism, and how sex- and age-related factors shape treatment effectiveness and safety. By narrowing scope and emphasising practical, multidisciplinary prevention strategies, this review aims to support clinicians in reducing recurrent events, disability, and mortality in very old patients. Future work should prioritise pragmatic trials, including those involving the oldest old and the use of standardised frailty metrics, to inform prevention decisions.

## 1. Introduction

Stroke represents a significant cause of mortality and disability [1]. Its impact is particularly pronounced in the elderly, who are more vulnerable because of the concurrence of factors such as senescence changes and a higher prevalence of comorbidities [1], including atrial fibrillation (AF), arterial hypertension (AH), diabetes mellitus (DM), heart failure (HF), and coronary artery disease (CAD) [2,3,4]. It is common knowledge that an effective prevention in this population requires blood pressure (BP) and metabolic control, smoking cessation, and oral anticoagulation therapy (OAC) if patients are affected by AF [5,6].

**Figure 1:**
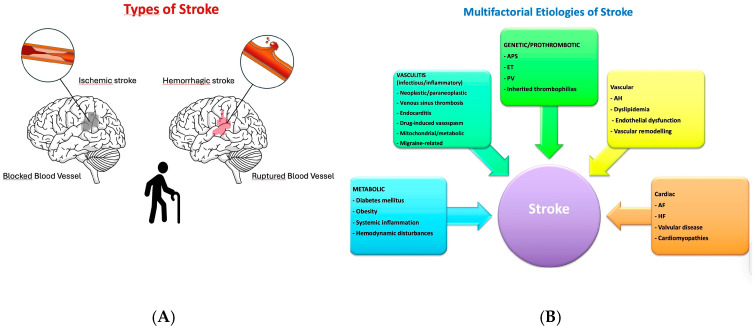
(**A**) Stroke Classification. This figure illustrates the main categories of stroke, distinguishing between ischemic and hemorrhagic subtypes. Ischemic strokes are further divided based on aetiology, including large-artery atherosclerosis, cardioembolism, and small-vessel occlusion. Hemorrhagic strokes are categorised into intracerebral and subarachnoid types, emphasising the underlying pathophysiological differences. (**B**) Multifactorial Etiologies of Stroke. In addition to conventional vascular risk factors (hypertension, dyslipidemia, endothelial dysfunction), major contributors include cardiac disorders (atrial fibrillation, heart failure, valvular disease, cardiomyopathies), metabolic derangements (diabetes mellitus, obesity, systemic inflammation, hemodynamic disturbances), and genetic or acquired prothrombotic states. A broad spectrum of atypical causes—such as vasculitis, haematological disorders, neoplastic/paraneoplastic mechanisms, venous sinus thrombosis, infective endocarditis, drug-induced vasospasm, mitochondrial/metabolic syndromes, and migraine-related vasculopathy—further underlines that stroke represents the final common pathway of multiple, often overlapping, pathogenic processes. Abbreviations: AF: Atrial fibrillation, AH: Arterial hypertension, APS: Antiphospholipid antibody syndrome, DM: Diabetes mellitus, ET: Essential thrombocythemia, FI: Frailty Index, HF: Heart failure, OAC: Oral anticoagulation.

Clinically, stroke is classified into two main forms, known as ischemic and hemorrhagic (Figure 1A) [7,8,9]. Ischemic stroke, the most common type, results from thrombosis, embolism, or systemic hypoperfusion, often linked to cardiac arrhythmias, acute myocardial infarction (AMI), or HF. Hemorrhagic stroke, including intracerebral haemorrhage (ICH) and subarachnoid haemorrhage (SAH), occurs due to vessel rupture, frequently caused by AH, coagulopathies, cerebral amyloid angiopathy, or vascular malformations. SAH, often triggered by aneurysmal rupture, results in the rapid dispersion of blood into the cerebrospinal fluid, thereby increasing intracranial pressure.

It is essential to identify the aetiology of stroke according to the Toast classification, which includes five subtypes: atherosclerosis of the great arteries (LAA), cardioembolic, small-vessel occlusion (SVO), stroke of undetermined cause (e.g., cryptogenic stroke) and stroke of ‘other’ cause in light of the fact that on the basis of this classification an appropriate therapy should be required [10].

However, it is worth noting that stroke has been recently recognised as a neurological disease after the recent ICD classification update, although it remains strongly conditioned by cardiovascular (CV) risk factors and CV comorbidities [11,12,13].

This condition is heterogeneous and multifactorial, resulting from vascular, cardiac, metabolic, genetic, and environmental factors, which must be considered [14]. Frailty is a multidimensional clinical syndrome characterised by reduced physiological reserve, impaired homeostatic mechanisms, and increased vulnerability to stressors. It reflects the cumulative effect of comorbidities, functional limitations, cognitive impairment, and metabolic dysregulation, resulting in a higher risk of adverse outcomes, including falls, disability, hospitalisation, and mortality. In patients with cardiovascular disease (CVD), frailty interacts with polypharmacy, multimorbidity, and age-related vascular changes, influencing treatment tolerance and therapeutic decision-making. Its identification is therefore essential in elderly individuals at risk of stroke, as it supports tailored strategies for primary and secondary prevention [14,15].

Structural cardiac disorders (including valvular disease, HF, and cardiomyopathies) and acquired or inherited prothrombotic states further increase susceptibility to cerebral ischemia [16,17,18,19].

Beyond these established mechanisms, a broad spectrum of less common etiologies underscores the complexity of stroke pathogenesis [20,21,22,23]. Infectious or inflammatory vasculitis can precipitate cerebrovascular events [24,25,26,27]. Haematological disorders, including antiphospholipid antibody syndrome, essential thrombocythemia, polycythemia vera, and rare inherited thrombophilias, contribute to hypercoagulability [28,29]. Neoplastic processes may promote cerebral ischemia via cancer-associated coagulopathy or paraneoplastic syndromes [30,31]. Other atypical causes include cerebral venous sinus thrombosis, septic or marantic endocarditis, drug-induced vasospasm, mitochondrial or metabolic syndromes, and migraine-related vasculopathy [32,33,34]. This broad etiologic spectrum emphasises that stroke should be regarded not as a single disease, but as the final common manifestation of multiple, often overlapping, pathogenic pathways [20,35] (Figure 1B).

In light of this complex interplay, an integrated, multidisciplinary approach is required, particularly in older adults [36]. Beyond acute management, which is outside the scope of this review, it is essential to include neurologists alongside cardiologists, geriatricians, internal medicine physicians, radiologists, emergency medicine professionals, rehabilitation specialists, surgeons and other specialists in the preventive and long-term care pathways [37]. Additionally, nurses, caregivers, and family members play a pivotal role in patient care, being essential to long-term care and recovery [38].

This review aims to provide a focused analysis of primary and secondary prevention of ischemic stroke in the elderly, with particular attention to the oldest old. Special emphasis is placed on the role of frailty indices in guiding risk–benefit decisions and on the implementation of practical care pathways. Within this framework, the review centres on the frail, elderly patient with a history of ischemic stroke who is managed in the post-acute phase within non-neurological care settings, reflecting a real-world scenario in which prevention strategies must be adapted to complex comorbidity profiles, shifting care environments, and persistent unmet clinical needs.

## 2. Epidemiology of Stroke in the Elderly with CVD

**Figure 2:**
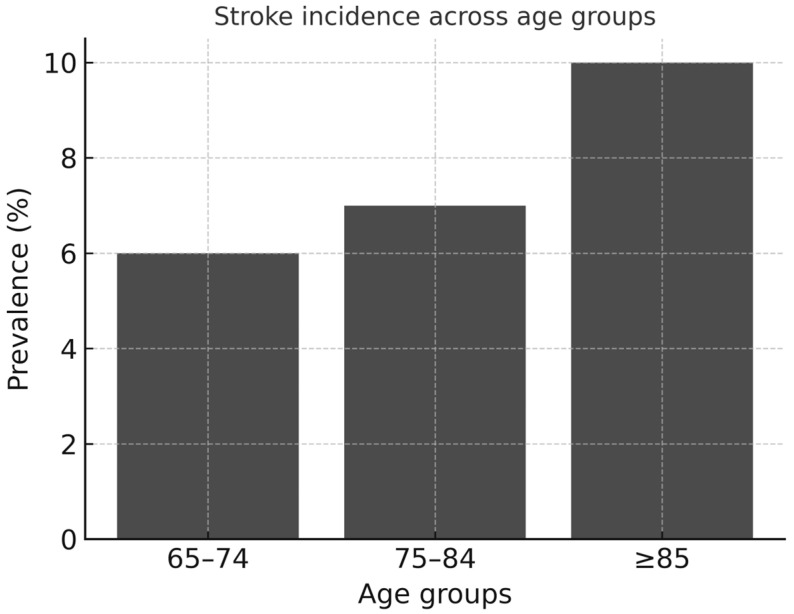
Stroke epidemiology in older adults with cardiovascular disease. The left panel illustrates the progressive increase in incidence with advancing age, exceeding 10% in patients aged 85 years and older.

It is well established that stroke incidence notably increases with age, imposing a substantial burden on elderly populations, particularly those with underlying CVD [9,39] (Figure 2).

Among CV comorbidities, AF, CAD, and HF are the key drivers of stroke in older adults. These conditions not only increase baseline stroke incidence (with primary prevention relevance) but also significantly affect recurrence rates and long-term outcomes (with secondary prevention relevance) [40,41,42,43].

Gender differences add further complexity. Frailty further amplifies this risk, as it is associated with multimorbidity, polypharmacy, and impaired resilience to vascular insults. Women, who generally have a longer life expectancy, experience a higher overall prevalence of stroke and greater disability after the event, partly due to delayed access to optimal prevention and rehabilitation strategies [9,35,40,41,43,44].

Despite clear guideline recommendations, effective preventive therapies remain underutilised in frail older patients due to concerns about bleeding, comorbidity, or life expectancy [45].

Women tend to experience greater post-stroke disability than men, in part due to a higher prevalence of post-stroke depression, which can impede rehabilitation efforts [45].

Notably, cardioembolic stroke is more frequent in individuals over 80 years old, largely due to the higher prevalence of AF in the elderly [3]. Women demonstrate a higher burden of post-stroke disability compared with men, with observational analyses reporting approximately 30% greater odds of functional impairment and long-term dependency [44]. In parallel, cardioembolic stroke becomes increasingly prevalent with advanced age: in individuals aged ≥80 years, the cardioembolic subtype accounts for nearly 36–40% of ischemic strokes, primarily driven by the sharply rising incidence of atrial fibrillation in this age group. These data reinforce the importance of risk factor optimisation and anticoagulation strategies in older adults, particularly women, who experience disproportionately worse post-stroke outcomes [40].

## 3. Frailty and Decision-Making

Stroke is a multifactorial disease with numerous potential causes that may interact in complex ways. In older adults, this complexity is amplified by age-related frailty, comorbidities, and polypharmacy [46,47]. Ageing also promotes vascular changes such as loss of arterial elasticity and increased stiffness, which increase susceptibility to cerebrovascular events and increased stiffness [48,49]. The interplay of frailty and comorbid conditions not only increases the risk of stroke but also influences clinical presentation, diagnostic accuracy, therapeutic response, and long-term outcomes. Frailty has been increasingly recognised as an independent determinant of prognosis, underscoring the importance of structured assessments—such as the Clinical Frailty Scale (CFS), the Fried criteria, the Frailty Index (FI), and the Edmonton Frail Scale (EFS), to inform treatment decisions and care planning [50] (Table 1).

Significantly, chronological age alone should not drive therapeutic choices, as two patients of the same age may differ profoundly in functional reserve and tolerance to treatment.

In clinical practice, frailty-informed decision-making prioritises risk stratification and focuses on major modifiable factors, such as blood pressure, lipids, and AF, as the cornerstone of prevention strategies. Effective risk factor management should not be withheld from frail individuals: AH, dyslipidaemia, and smoking cessation require tailored but active intervention, complemented by lifestyle measures such as regular physical activity and adherence to a heart-healthy diet. Critically, frailty alone should not preclude OAC. A patient-centred balance of risks and benefits is recommended, as the protective effect against stroke usually outweighs the bleeding risk, provided that close monitoring and fall-prevention strategies are implemented [6,51]. Despite this, OAC remains underutilised in frail elderly patients due to concerns about bleeding and falls.

Beyond pharmacology, frailty assessment supports shared decision-making with patients and caregivers, helping to align treatment intensity with realistic care goals and expected life expectancy. Rather than promoting therapeutic nihilism, frailty evaluation provides a framework for personalised, pragmatic choices that balance efficacy, safety, and quality of life in elderly patients with cardiovascular disease (CVD) at risk of stroke.

**Table 1:** Validated frailty assessment tools in elderly patients with stroke and CVD.

Tool	Key Components	Scoring/Definition	Clinical Use
CFS	Global clinical judgement integrating comorbidity burden, cognitive status, mobility, and functional independence [52]	9-point scale (1 = very fit; 9 = terminally ill)	Rapid bedside screening tool applicable in acute and subacute stroke settings; supports decisions on treatment intensity (e.g., anticoagulation eligibility, blood pressure targets) and informs discharge planning and level of care [53]
Fried Frailty Phenotype	Unintentional weight loss, muscle weakness, self-reported exhaustion, slow gait speed, low physical activity [54]	Frailty defined by ≥3 criteria; pre-frailty by 1–2 criteria	Identifies patients with predominant physical frailty and higher vulnerability to disability and falls; mainly applied in outpatient care and population-based risk stratification [54]
FI	Accumulation of health deficits across comorbidities, functional impairment, cognitive decline, and psychosocial domains [55,56]	Ratio of present deficits to total deficits assessed (range 0–1)	Provides a comprehensive estimate of biological vulnerability; primarily used for prognostic evaluation, long-term risk assessment, and advanced care planning [55,56]
EFS	Multidimensional assessment including cognition, mood, functional performance, social support, nutritional status, and medication burden [57,58]	17-point scale (0 = not frail; 17 = severely frail)	Structured and feasible instrument in routine clinical practice; supports individualised therapeutic decisions and multidisciplinary care coordination [57,58].

Abbreviations: CFS: Clinical Frailty Scale; FI: Frailty Index; EFS: Edmonton Frail Scale. These recommendations reflect the authors’ interpretation of the available evidence and clinical experience, and are supported by the references provided.

## 4. Primary Prevention of Stroke in the Elderly

In elderly and frail patients, risk factor management is crucial to reduce stroke incidence, but treatment strategies must be tailored to frailty, comorbidities, and life expectancy (Table 2). Conventional risk factors, including AH, dyslipidemia, DM, AF, and smoking, interact with age-related vascular changes such as arterial stiffness, endothelial dysfunction, systemic inflammation, and polypharmacy, thereby amplifying cerebrovascular risk [43,46,47,48,49].

Cardiac disorders, including HF, valvular disease, and cardiomyopathies, further predispose to ischemic events by impairing cerebral perfusion and increasing embolic risk [59].

Hypertension. AH, defined as systolic blood pressure ≥ 140 mmHg and/or diastolic blood pressure ≥ 90 mmHg, remains one of the leading modifiable risk factors for ischemic stroke [60], contributing to approximately one-quarter of stroke cases [61]. While BP lowering is a cornerstone of prevention, abrupt reductions may be harmful, particularly in patients with carotid stenosis [62]. In the frail elderly, aggressive BP reduction may cause orthostatic hypotension and falls, so therapeutic goals should be less stringent and titration should be gradual [63,64].

Dyslipidemia. Dyslipidemia accelerates atherosclerosis, the leading cause of CVD after 65 years of age. Risk stratification should guide the intensity of lipid-lowering therapy (LLT). High CV risk in elderly patients is often linked to long-standing DM, established CVD, familial hypercholesterolemia, or elevated coronary calcium scores. Statins are well established in secondary prevention, including in those over 75, but their role in primary prevention in this age group is debated. Evidence remains limited as older and frail patients were underrepresented in trials [65,66,67,68,69,70,71]. Observational data suggest possible benefit in high-risk elders [72], but safety concerns—polypharmacy, hepatic dysfunction, and myopathy—are particularly relevant [70,73]. Guidelines therefore recommend an individualised initiation of lipid-lowering therapy, often starting at low doses (e.g., atorvastatin 10–20 mg/day, rosuvastatin 5–10 mg/day, or simvastatin 10–20 mg/day), with careful up-titration based on tolerance, frailty status, and comorbidity burden [68,71,74,75]. LDL-C targets remain the same as in younger adults [76,77,78,79]. However, the role of statins in elderly patients requires careful clinical consideration [70]. Evidence from secondary prevention trials supports statin therapy in older adults with established atherosclerotic CVD, demonstrating meaningful reductions in recurrent stroke and major vascular events. Nonetheless, data on primary prevention beyond the age of 75 remain limited because frail and elderly individuals are under-represented in randomised studies [70]. Clinical decision-making must therefore integrate comorbidity burden, polypharmacy, functional reserve, and estimated life expectancy [70]. Potential disadvantages include statin-associated myopathy, hepatic dysfunction, and drug–drug interactions—risks that may be amplified in frail older patients. For this reason, guidelines favour an individualised approach, often beginning with low-dose regimens and gradual titration. In selected high-risk patients who are unable to achieve LDL-C targets or are statin-intolerant, non-statin therapies such as ezetimibe or PCSK9 inhibitors may be appropriate adjuncts [80]. Overall, age alone should not exclude lipid-lowering therapy, but treatment intensity should be tailored to biological age, frailty phenotype, and patient priorities [70]. In asymptomatic carotid stenosis > 50%, a comprehensive strategy including statins, antiplatelets, and smoking cessation is indicated [78,81]; for stenosis > 70%, referral to a vascular neurologist is recommended [82].

Diabetes and metabolism. Insulin resistance is associated with increased recurrence and poorer outcomes [83], and the triglyceride–glucose (TyG) index has been proposed as a marker of recurrence risk [84]. Tight glycaemic control reduces CV risk [81,85], but in frail elderly patients, HbA1c targets should be more permissive than the standard 7% to avoid hypoglycaemia [86,87].

Lifestyle. Smoking cessation, diet optimisation, and physical activity remain effective preventive strategies; however, in frail patients, interventions must be realistic and tailored to their functional capacity.

Cardiac comorbidities. AF is considered one of the strongest risk factors for ischemic stroke [43]. HF and CAD contribute to the vulnerability by worsening perfusion and increasing the risk [59]. OAC is the most effective preventive intervention, with Direct Oral Anticoagulants (DOACs) preferred over warfarin due to lower intracranial bleeding [88]. Frailty alone should not preclude anticoagulation, but treatment requires careful assessment of renal function, drug interactions, fall risk, and closer follow-up [89]. In cases of concomitant CAD requiring antiplatelet therapy, combined regimens should be short and individualised.

Antiplatelet therapy. Antiplatelet agents are not routinely recommended for primary prevention in the elderly due to the risk of bleeding [90,91,92]. According to the AHA guidelines, aspirin use for primary prevention is recommended at Class III patients over 70 years of age.

**Table 2:** Frailty-Adapted Approaches to Primary Stroke Prevention in Older Adults.

	Standard Recommendation	Frailty-Informed Adaptation
Hypertension	BP lowering reduces stroke risk; strict targets recommended [2,93]	More flexible targets; avoid abrupt reduction; monitor for orthostatic hypotension and falls [2,63,93].
Dyslipidemia/LLT	Statins reduce CV risk; same LDL-C targets as in younger adults [94]	Limited evidence > 75 yrs; initiate only if high CV risk; start low doses; weigh benefit vs. polypharmacy, comorbidity, life expectancy [94]
Diabetes/metabolism	HbA1c ~7% as general target	More lenient targets (>7%) may be safer; minimise hypoglycaemia and treatment burden [95,96,97]
Lifestyle factors	Smoking cessation, healthy diet, regular exercise [81]	Interventions must be feasible and aligned with functional capacity; focus on achievable lifestyle changes [81].
Atrial fibrillation/OAC	DOACs preferred over warfarin; anticoagulation is standard [98]	Frailty not a contraindication; closer monitoring needed; assess renal function, falls, drug interactions; shared decision-making with caregivers [3,6,98]
Antiplatelet therapy	Considered for primary prevention in selected younger high-risk patients [99,100]	Not recommended >70 yrs; AHA Class III against aspirin due to bleeding risk; avoid in frail elderly [99,100]

Abbreviations: BP = Blood Pressure; LLT = Lipid-Lowering Therapy; LDL-C = Low-Density Lipoprotein Cholesterol; HbA1c = Haemoglobin A1c; OAC = Oral Anticoagulation; DOACs = Direct Oral Anticoagulants; CV = Cardiovascular; AHA = American Heart Association.

## 5. Secondary Prevention of Stroke in Elderly Patients with CVD

Secondary stroke prevention in elderly patients with CVD requires a personalised, evidence-based strategy that judiciously weighs the protective effects of antithrombotic treatment against the potential for bleeding complications [81,90,101]. Advances in imaging modalities, long-term cardiac monitoring, and targeted pharmacological interventions have enhanced the ability to personalise treatment strategies, thereby reducing stroke recurrence and improving overall patient outcomes [81].

In frail elderly patients, secondary prevention must always be contextualised within functional status, comorbidity burden, polypharmacy, and life expectancy, with the primary goal of reducing recurrence while preserving safety and quality of life (Table 3).

**Figure 3:**
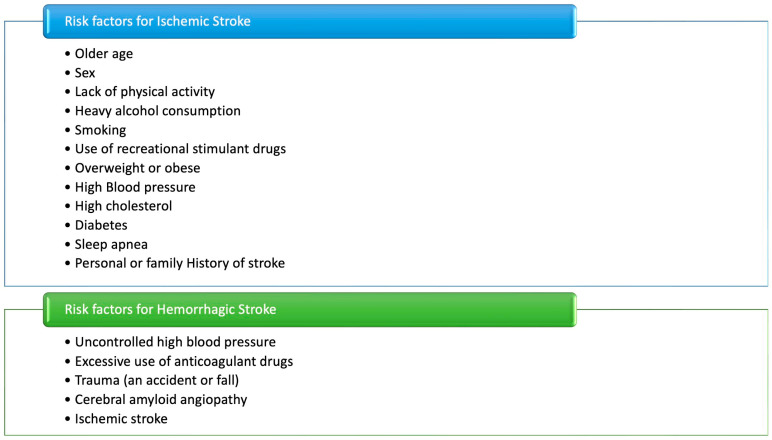
Risk factors for ischemic and hemorrhagic stroke. Vascular risk factors and lifestyle behaviours promote ischemic stroke, while hemorrhagic stroke is mainly driven by uncontrolled hypertension, anticoagulant use, trauma, and cerebral amyloid angiopathy.

An effective strategy for secondary stroke prevention must be tailored to the underlying aetiology, as determined by TOAST classification criteria [102]. Key components include the management of modifiable risk factors, antithrombotic therapy, and lipid-lowering treatment with high-intensity statins [90] (Figure 3).

### 5.1. Risk Factor Management

Optimal blood pressure control is an essential strategy for secondary prevention of stroke; however, aggressive reductions in the acute phase should be avoided to prevent cerebral hypoperfusion [81].

According to the most recent guidelines, blood pressure should be lowered to the lowest safely achievable level, with a preferred systolic target below 140 mmHg in cases where the optimal range of 120–129 mmHg (Class I recommendation) cannot be tolerated [81,103]. This applies particularly to patients with pre-treatment symptomatic orthostatic hypotension, individuals aged 85 years or older (Class IIa), or those with a moderate to severe degree of frailty (Class IIb) [62,81,104].

Another suggestion for treatment strategy confirmed in the recent guidelines is to start with monotherapy in those patients.

Additionally, the ESH Guidelines establish different targets for patients aged over 80 years, based on their functional capacity and autonomy status [104].

Although the optimal blood pressure targets remain a subject of ongoing debate, current clinical guidelines advocate maintaining values below 140/90 mmHg [105,106,107]. However, there is a general consensus of opinion over the fact that in individuals with T2DM, a more stringent target of less than 130/80 mmHg is typically recommended to reduce CV risk. Resumption of antihypertensive therapy is generally advised within 24 h of stroke onset, with careful titration to minimise the risk of recurrent stroke or hemodynamic instability [108,109].

Glycaemic control and smoking cessation are also critical. In patients with T2DM, a target HbA1c of approximately 7% is recommended, with more relaxed thresholds in frail individuals to minimise hypoglycaemia risk. Lifestyle measures—including dietary optimisation, regular physical activity, and weight control—should complement pharmacological treatment [110].

For frail elderly patients, lifestyle recommendations should be realistic and tailored to their functional reserve, with an emphasis on safety and feasibility rather than adhering to strict numerical targets.

In elderly patients, prolonged cardiac rhythm monitoring with an implantable loop recorder is particularly valuable for detecting subclinical AF, which is a major cause of cardioembolic stroke [3]. Other significant risk factors, including AH, T2DM and smoking, contribute predominantly to small-vessel disease and require aggressive management [110].

### 5.2. Antithrombotic Therapy

The timing and choice of antithrombotic therapy depend on stroke aetiology and associated risks. In patients already on anticoagulation at the time of stroke, temporary discontinuation may be necessary, particularly if thrombolysis is being considered [111,112].

For cardioembolic stroke, DOACs are preferred [113]. A simple, severity-based timing approach can be adopted (e.g., starting around day 3 after a minor stroke, day 6 after a moderate stroke, and day 12 after a severe stroke), balancing the risk of hemorrhagic transformation and infarct size [113]. Where bleeding risk is high, initiation can be deferred within the 2–14-day window with careful BP control. Frailty alone should not delay or preclude anticoagulation, but warrants individualised monitoring and fall-risk mitigation [81].

It has been suggested that early DOAC initiation (≤4 days after ischemic stroke onset) may reduce the risk of recurrent stroke and intracranial haemorrhage compared to later initiation (≥5 days) [114,115]. However, this benefit was not statistically significant at 90 days [114,115]. However, the optimal anticoagulation timing in this subgroup remains debated.

In patients with recent AMI and left ventricular (LV) dysfunction (ejection fraction < 40%), short-term warfarin therapy (up to 3 months) may be considered, even in the absence of intracardiac thrombi, to mitigate thromboembolic risk [116,117]. The role of thrombophilia screening in stroke prevention remains uncertain and requires further investigation.

Antiplatelet therapy remains the standard approach for non-cardioembolic stroke. Aspirin (80–365 mg) is the first-line treatment, with clopidogrel or P2Y12 inhibitors as alternatives. Dual-antiplatelet therapy (DAPT) with aspirin and clopidogrel may be initiated within 24 h of stroke onset and continued for 21 days, particularly in patients with CAD or high-risk transient ischemic attack (TIA) [118]. However, long-term DAPT is generally not recommended due to an increased risk of bleeding without additional mortality benefits [119,120].

In cases of hemorrhagic infarction, antiplatelet or anticoagulant therapy should be withheld for one to two weeks until clinical stabilisation [121]. In selected patients with mild and stable hemorrhagic transformation, low-dose aspirin (e.g., 75–100 mg/day) may be continued under careful clinical and imaging surveillance [122]. It has been widely accepted that in patients at high risk due to intracranial atherosclerosis, aggressive medical therapy has proven more effective than intracranial stent placement in reducing the risk of recurrent cerebrovascular events [123,124,125]. In frail elderly patients, the use of antiplatelet or anticoagulant therapy should always be guided by a careful evaluation of bleeding risk, comorbidities, and expected benefit, avoiding overtreatment.

### 5.3. Lipid Management and Statin Therapy

Lipid-lowering therapy is a fundamental aspect of secondary stroke prevention, particularly in patients with concomitant CVD [81,101,126]. High-intensity statin therapy (e.g., atorvastatin 80 mg) or PCSK9 inhibitors should be considered to achieve an LDL cholesterol target of <55 mg/dL, particularly in patients with coexisting cardiac disease [127]. It has been pointed out that achieving this target has been associated with a 28% reduction in stroke recurrence [127]. In the frail elderly, treatment should be individualised: the benefit of intensive LDL-C lowering must be balanced against polypharmacy, drug–drug interactions, and risk of adverse effects such as myopathy or hepatic dysfunction. Age alone should not exclude therapy; however, treatment intensity should be tailored to match overall frailty and life expectancy.

### 5.4. Carotid Disease and Revascularisation

Carotid endarterectomy (CEA) [128] is preferred over carotid artery stenting (CAS) in elderly patients, particularly when performed within two weeks of stroke onset in cases of carotid stenosis > 70%. The choice between CEA and CAS in elderly patients remains complex. Current AHA/ASA guidelines recommend CEA over CAS in patients aged ≥ 70 years (Class IIa, Level of Evidence B), largely due to a higher periprocedural stroke risk reported with CAS in this age group [15]. However, CEA has been associated with an increased incidence of perioperative cardiac complications in older adults, highlighting important trade-offs [129]. Therefore, treatment should be individualised, and CAS may represent a viable option in elderly patients who are poor surgical candidates or at elevated surgical risk [129,130,131]. For patients with moderate carotid stenosis (50–69%), surgical intervention is generally not indicated, as medical therapy alone is sufficient to prevent stroke recurrence. In frail patients, decisions on revascularisation should carefully consider perioperative risk, functional autonomy, and overall prognosis, with a preference for best medical therapy when surgical benefit is marginal.

**Table 3:** Frailty-Adapted Approaches to Secondary Stroke Prevention in Older Adults.

	Standard Recommendation	Frailty-Informed Adaptation
Lifestyle interventions	Smoking cessation, diet optimisation, physical activity [132]	Interventions must be realistic and adapted to functional status; prioritise safety and quality of life rather than strict targets
Hypertension	Target SBP < 140 mmHg (optimal 120–129 mmHg if tolerated); early resumption of therapy after stroke onset [133]	More flexible targets in ≥85 yrs or frail patients; avoid aggressive lowering; start with monotherapy; prioritise tolerance and fall prevention [133]
LLT	High-intensity statin ± PCSK9i to achieve LDL < 55 mg/dL	Individualise based on frailty, life expectancy, and polypharmacy; start low dose; monitor closely for adverse effects; focus on high-risk patients (established CVD) [134]
Glycaemic control	HbA1c ≈ 7% in patients with T2DM	Relaxed HbA1c targets (>7%) in frail patients to minimise hypoglycaemia; individualise based on functional reserve and comorbidities [135]
Antiplatelet therapy	Aspirin or clopidogrel for non-cardioembolic stroke; short-term DAPT (21 days) in selected patients [81,136,137]	Careful bleeding risk assessment; avoid long-term DAPT; de-escalate therapy if risk > benefit; frailty and comorbidities guide intensity [81,136,137]
Anticoagulation for AF	DOACs preferred; timing based on stroke severity (≈day 3, 6, 12) [138]	Frailty not a contraindication; closer monitoring of renal function, adherence, fall risk; involve caregivers in shared decision-making [3,6,64,136,139,140,141,142]
Carotid revascularisation	CEA within 2 weeks for symptomatic > 70% stenosis; CAS generally avoided in elderly [143,144,145]	In frail patients, carefully weigh perioperative risk vs. benefit; often best medical therapy preferred over intervention when prognosis or autonomy is limited [146,147]

In frail elderly patients, secondary stroke prevention requires the same therapeutic approach as in the general population. Still, intensity and timing must be tailored to functional status, comorbidities, and life expectancy. Frailty is not a contraindication, but a key determinant of individualised decision-making. Abbr: AF: Atrial fibrillation, CAS: Carotid artery stenting, CEA: Carotid endarterectomy, CVD: Cardiovascular disease, DAPT: Dual antiplatelet therapy, DOACs: Direct oral anticoagulants, HbA1c: Glycated haemoglobin; LDL: Low-density lipoprotein; LLT: Lipid-lowering therapy; PCSK9i: Proprotein convertase subtilisin/kexin type 9 inhibitors; SBP: Systolic blood pressure; T2DM: Type 2 diabetes mellitus.

## 6. Diagnostic Work-Up for Secondary Prevention

Diagnostic evaluation after ischemic stroke plays a pivotal role in shaping secondary prevention strategies, particularly in frail elderly patients [148]. In this population, clinical presentation is frequently atypical, physiological reserve is reduced, and comorbidity burden may affect both diagnostic yield and test feasibility [149]. Understanding how diagnostic pathways must be adapted to cognitive status, mobility limitations, sarcopenia, renal function, and tolerance to invasive procedures is essential to guide personalised anticoagulation, antiplatelet therapy, lipid management, and arrhythmia detection. Therefore, etiologic work-up must not simply replicate standard approaches but incorporate frailty status and life expectancy to avoid overtesting, undertesting, or inappropriate treatment intensification [148,149,150,151,152].

These diagnostic considerations are particularly prominent in elderly patients with cardiovascular disease (CVD), in whom establishing the aetiology of stroke is particularly challenging, since clinical features may overlap with comorbidities [17]. From a neurological perspective, frailty modifies ischemic lesion patterns, increases the rate of silent infarcts, and is associated with delayed recognition due to cognitive and behavioural fluctuations. These factors must be considered when interpreting neuroimaging findings and planning [153] follow-up strategies. In practical terms, imaging results in frail patients may carry a different prognostic weight than in fitter individuals, and this has direct implications for long-term stroke recurrence prevention [149,151]. Furthermore, atypical or nonspecific presentations, such as confusion, fatigue, or altered mental status, can be easily misinterpreted as dementia or delirium [154]. Meanwhile, concomitant CV symptoms (e.g., chest pain, dyspnea, syncope) may further obscure the underlying neurological event [17].

For secondary prevention, the priority is to identify the causal mechanism of stroke, as management differs according to aetiology. In frail older individuals, this step becomes particularly meaningful, as the etiologic definition of stroke directly informs treatment intensity. Confirming a cardioembolic source, for example, may justify anticoagulation even in the presence of falls or cognitive impairment, whereas recognising a small-vessel mechanism might encourage a more conservative approach to reduce bleeding risk and drug interactions. In practice, diagnostic clarification supports individualised choices and avoids therapeutic extremes [155]. This diagnostic-to-therapeutic transition is fundamental, as tailoring treatment to this classification is crucial to prevent recurrence. This linkage between diagnosis and prevention begins in the acute setting, where the diagnostic pathway already determines key therapeutic decisions in elderly patients [153,155,156,157,158]. The diagnostic pathway initiated during the acute phase also influences secondary prevention decisions in elderly patients. Early brain imaging affects timing of anticoagulation in cardioembolic stroke, while acute vascular imaging informs the need for carotid revascularisation. Likewise, echocardiographic evaluation performed during hospitalisation enables risk stratification for left ventricular thrombus, guiding anticoagulation intensity and duration. In frail individuals, the feasibility and tolerance of early MRI, CTA, or transesophageal echocardiography may be limited, requiring pragmatic adaptations without compromising diagnostic accuracy [159].

Imaging retains a central role, not to establish the initial diagnosis but to define stroke subtype and guide preventive strategies. MRI—particularly diffusion-weighted imaging (DWI) and perfusion sequences—offers high sensitivity for small cortical and subcortical infarcts, microangiopathy, and silent infarcts, all of which are frequent in the elderly [160]. CT angiography (CTA) and CT perfusion (CTP) provide additional insights into vascular anatomy and haemodynamics, supporting the identification of large-artery disease or hemodynamic compromise [161]. Advanced MRI paradigms are increasingly recommended to refine etiologic classification and stratify recurrence risk [162].

When a stroke is cryptogenic, identifying the potential cause is crucial [163]. Transesophageal echocardiography (TEE) is particularly valuable for detecting left atrial thrombi, valvular vegetations, and complex aortic plaques [81]. Transthoracic echocardiography (TTE) complements this assessment by evaluating cardiac function and structural abnormalities. In parallel, prolonged cardiac monitoring is strongly advised in elderly patients, since paroxysmal AF is highly prevalent and frequently missed on routine ECG [164].

From a frailty perspective, the extent of the diagnostic work-up must be balanced with feasibility and safety (Table 4). Some elderly patients may not tolerate prolonged MRI protocols, invasive echocardiography, or repeated contrast studies. Nonetheless, a pragmatic yet comprehensive etiologic assessment remains indispensable, as it enables clinicians to tailor secondary prevention and reduce the risk of recurrence.

**Table 4:** Diagnostic Modalities for Secondary Stroke Prevention in the Elderly: Strengths, Limitations, and Clinical Role.

Modality	Main Strengths	Limitations in Frail Elderly	Principal Clinical Role
CT (non-contrast)	Widely available; essential to exclude haemorrhage [165]	Low sensitivity for small/early ischemia; radiation exposure [165]	First-line tool; baseline evaluation to guide further work-up [165]
CT Angiography (CTA)/CT Perfusion (CTP)	Provides vascular anatomy and perfusion; supports detection of LAA or hemodynamic compromise [166]	Requires contrast (risk of nephropathy); higher radiation burden [166]	Etiologic classification of large artery disease; assessment of vascular reserve [166]
MRI (DWI/FLAIR ± perfusion, MRA)	Highest sensitivity for ischemia, small-vessel disease, silent infarcts; prognostic value; useful for etiologic stratification [167,168]	Limited availability; long acquisition; contraindicated in implants; may be poorly tolerated in frail/cognitively impaired [167,168]	Defining ischemic subtype (SVO vs. cardioembolic vs. cryptogenic); prognosis [167,168]
Carotid and transcranial ultrasound	Non-invasive, bedside, radiation-free; identifies extracranial stenosis and microembolic signals	Operator-dependent; limited sensitivity for intracranial disease	Screening of carotid stenosis; follow-up and monitoring
Transthoracic echocardiography (TTE)	Non-invasive; evaluates LV thrombi, EF, cardiomyopathies	Lower yield for atrial thrombi or aortic plaques	Initial cardiac evaluation, especially in patients unfit for TEE
Transoesophageal echocardiography (TEE)	Gold standard for detecting atrial thrombi, valvular vegetations, complex aortic plaques, PFO	Semi-invasive; requires sedation; less feasible in frail or cognitively impaired elderly	Definitive search for embolic sources; critical in cryptogenic stroke
Prolonged cardiac monitoring	Detects paroxysmal AF, frequent in elderly	Device-related burden; adherence difficulties	Etiologic clarification in cryptogenic stroke; guiding OAC decisions

In elderly and frail patients, etiologic work-up after stroke should be comprehensive but adapted to feasibility. Neuroimaging and cardiac investigations are indispensable to classify stroke subtype, but procedural burden and tolerance must guide a frailty-informed, patient-centred approach. Diagnostic evaluation in this population requires balancing speed, accuracy, and feasibility. Abbreviations: AF: Atrial fibrillation; CTA: Computed tomography angiography; CTP: Computed tomography perfusion; CT: Computed tomography; DWI = Diffusion-weighted imaging; EF: Ejection fraction; FLAIR: Fluid-attenuated inversion recovery; LV: Left ventricle; MRA: Magnetic resonance angiography; MRI: Magnetic resonance imaging; OAC: Oral anticoagulation; PFO: Patent foramen ovale; SVO: Small-vessel occlusion; TEE: Transoesophageal echocardiography; TTE: Transthoracic echocardiograph.

## 7. Long-Term Stroke Management and Rehabilitation

### 7.1. Rehabilitation

After the acute phase, long-term stroke management in elderly patients with CVD focuses on rehabilitation, secondary prevention, and continuous monitoring of CV and neurological health [169]. Stroke rehabilitation aims to maximise functional recovery through a multidisciplinary approach incorporating physical, occupational, and speech therapy [81].

Indeed, it has been claimed that approximately 30% of stroke survivors experience persistent functional impairments, increasing the risk of falls, instability, and pressure ulcers [170].

### 7.2. Lifestyle Management

Nutritional optimisation, including sodium restriction (2–4 g/day), plays a crucial role in post-stroke recovery [171]. Sleep assessment, particularly for sleep apnea, is vital given that its high prevalence in this population and its association with worse outcomes [172].

**Figure 4:**
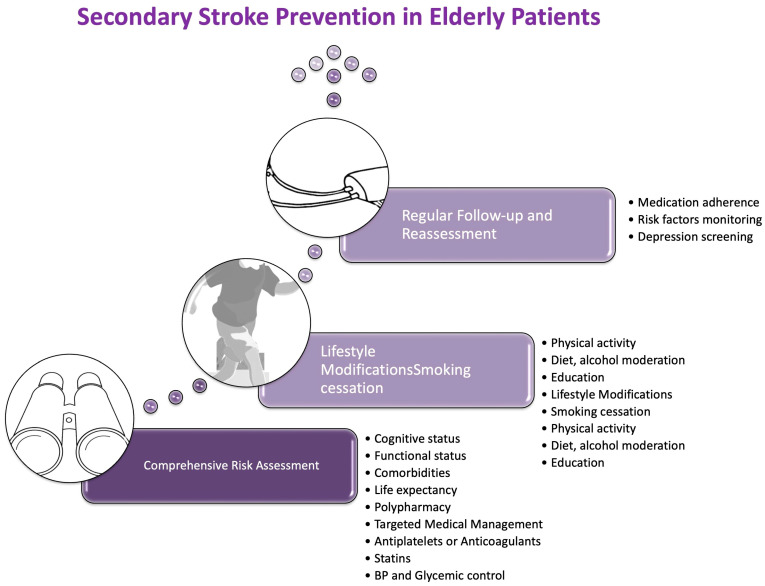
Secondary stroke prevention in elderly patients. Key strategies include comprehensive risk assessment, optimisation of medical therapy (antiplatelets or anticoagulants, statins, blood pressure and glucose control), and lifestyle interventions such as smoking cessation, physical activity, and dietary moderation. Regular follow-up supports adherence and addresses psychological factors.

Secondary prevention involves rigorous control of modifiable risk factors, such as AH, hyperlipidemia, and DM, alongside the management of underlying cardiac conditions (Figure 4).

### 7.3. Cognitive Support

Stroke also significantly exacerbates cognitive decline in elderly patients [173]. Evidence from randomised studies suggests that structured exercise combined with mental stimulation may improve cognitive function [173].

### 7.4. Psychosocial Support

Depression and anxiety are frequent after stroke, hampering recovery and adherence to treatment [174]. In many cases, care is improvised through family support or telemedicine, which may not always be feasible for elderly patients. Psychosocial support through family involvement, psychological care, and telemedicine-based interventions helps address these barriers and improves quality of life [76].

### 7.5. Cardiac Rehabilitation

Cardiac rehabilitation is particularly beneficial for patients with HF or CAD, as it reduces the risk of recurrent stroke and improves overall CV well-being [81].

## 8. Multidisciplinary Care and Coordination

Effective stroke management in the elderly with CVD requires a coordinated approach involving multiple healthcare professionals [175]. Neurologists, cardiologists, geriatricians, and rehabilitation specialists must collaborate to develop a comprehensive care plan tailored to the individual’s specific needs [173].

In a multidisciplinary team, each professional has a specific role that contributes to comprehensive care. Neurologists play a central role in the diagnostic process, selecting acute treatments, and planning secondary prevention. Cardiologists evaluate and manage AF, CAD and HF, which are frequent comorbidities in this setting. Geriatricians assess frailty, polypharmacy, and functional capacity, helping to tailor treatment strategies to the patient’s overall condition. Rehabilitation specialists and physiatrists design and supervise programmes aimed at motor and cognitive recovery, thereby supporting functional independence. Nutritionists provide dietary counselling and help control CV risk factors through nutritional interventions. Psychiatrists and psychologists assist in the management of depression, anxiety, and cognitive impairment, which often complicate the recovery process [36,38,76].

Close monitoring and early intervention can prevent complications and optimise recovery.

**Figure 5:**
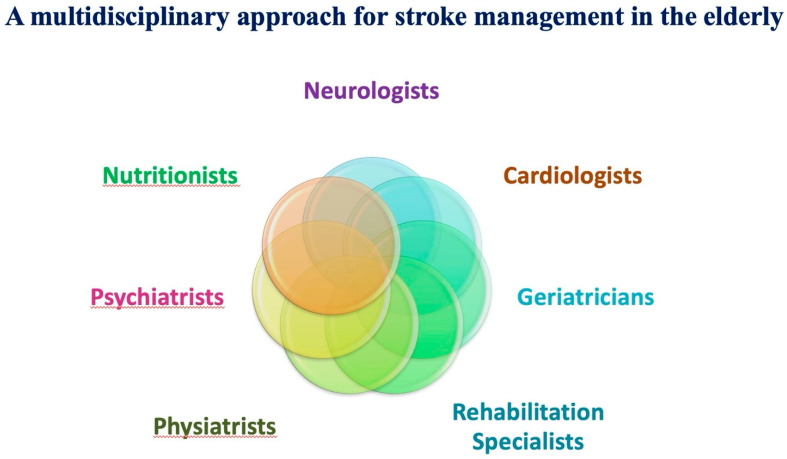
Multidisciplinary stroke management in the elderly. Optimal care requires collaboration among neurologists, cardiologists, geriatricians, rehabilitation experts, nutritionists, psychiatrists, and physiatrists to address the medical, functional, and psychosocial needs of older patients.

Furthermore, patient and caregiver education on the risks of stroke recurrence, the importance of medication adherence, and lifestyle modifications are essential components of long-term care [175] (Figure 5).

## 9. Ethical Considerations in the Management of Stroke in the Elderly

Management of stroke in elderly patients with CVD presents significant ethical challenges, especially concerning therapeutic decision-making and the consideration of end-of-life care preferences [176]. The decision to pursue aggressive interventions such as thrombolysis or mechanical thrombectomy in frail elderly patients with multiple comorbidities requires careful consideration of the risks and benefits. In cases of severe stroke or poor prognosis, discussions regarding palliative care and advanced directives should be initiated early to respect the patient’s autonomy and ensure that the care provided aligns with their values and preferences [177].

## 10. Conclusions

Stroke in elderly patients with CVD is a complex clinical challenge requiring an individualised, multidisciplinary approach. Timely identification and early therapeutic intervention are essential to limit the long-term consequences of stroke. Moreover, the implementation of evidence-based secondary prevention measures significantly decreases the likelihood of recurrence. Balancing stroke-specific therapies with CVD management is necessary to optimise outcomes in this vulnerable population. As the ageing population continues to grow, research into more effective treatments and management strategies for stroke in the elderly with CVD will remain a priority for clinicians and healthcare systems worldwide. Future efforts should prioritise the development of care models that integrate neurology, cardiology, geriatrics, and rehabilitation, ensuring continuity from the acute phase to long-term follow-up. Moreover, prospective studies specifically designed for elderly populations are urgently needed, as current evidence is often derived from subanalyses of trials that underrepresent this group.

Notably, the frailty-adjusted intervention frameworks presented in this review represent a synthesis of current evidence and clinical experience, supported by the literature cited throughout the manuscript, and are intended to provide a practical, transparent basis for informed clinical decision-making in real-world care settings.

Addressing these gaps is essential to provide equitable, effective, and patient-centred stroke care for the growing population of older adults with CVD.

## Data Availability

No new data were created or analyzed in this study. Data sharing is not applicable to this article.
